# Profiling of Plant Growth-Promoting Metabolites by Phosphate-Solubilizing Bacteria in Maize Rhizosphere

**DOI:** 10.3390/plants10061071

**Published:** 2021-05-27

**Authors:** Minchong Shen, Jiangang Li, Yuanhua Dong, Hong Liu, Junwei Peng, Yang Hu, Yang Sun

**Affiliations:** 1 CAS Key Laboratory of Soil Environment and Pollution Remediation, Institute of Soil Science, Chinese Academy of Sciences, Nanjing 210008, China; mcsh@issas.ac.cn (M.S.); yhdong@issas.ac.cn (Y.D.); liuhong@issas.ac.cn (H.L.); pengjw@issas.ac.cn (J.P.); yanghu6663@163.com (Y.H.); sunyang@issas.ac.cn (Y.S.); 2 University of Chinese Academy of Sciences, Beijing 100049, China; 3 School of Resources and Environment, Anhui Agricultural University, No. 130 Changjiang West Road, Hefei 230036, China

**Keywords:** maize growth, plant growth-promoting bacteria, phosphate-solubilizing bacteria, metabolomic, differential metabolites, plant growth-regulating molecules, biomarker, mechanism

## Abstract

Microbial treatment has recently been attracting attention as a sustainable agricultural strategy addressing the current problems caused by unreasonable agricultural practices. However, the mechanism through which microbial inoculants promote plant growth is not well understood. In this study, two phosphate-solubilizing bacteria (PSB) were screened, and their growth-promoting abilities were explored. At day 7 (D7), the lengths of the root and sprout with three microbial treatments, M16, M44, and the combination of M16 and M44 (Com), were significantly greater than those with the non-microbial control, with mean values of 9.08 and 4.73, 7.15 and 4.83, and 13.98 and 5.68 cm, respectively. At day 14 (D14), M16, M44, and Com significantly increased not only the length of the root and sprout but also the underground and aboveground biomass. Differential metabolites were identified, and various amino acids, amino acid derivatives, and other plant growth-regulating molecules were significantly enhanced by the three microbial treatments. The profiling of key metabolites associated with plant growth in different microbial treatments showed consistent results with their performances in the germination experiment, which revealed the metabolic mechanism of plant growth-promoting processes mediated by screened PSB. This study provides a theoretical basis for the application of PSB in sustainable agriculture.

## 1. Introduction

Maize (*Zea mays* L.) is the world’s third most important crop after wheat and rice, which together account for 94% of cereal consumption [1]. Widely planted in Africa, Mexico, Central America, and northeastern China, maize supplies nutrients necessary for human metabolic needs [2]. However, with urban expansion and the consequent reduction in cultivated land [3,4], agricultural production is threatened by several factors, including excessively intensive cultivation [5], continuous cropping for years [6], improper fertilization [7], and excessive use of chemical fertilizers [8], mainly due to inappropriate agricultural practices. Therefore, environmental challenges, such as soil acidification [9], imbalances in nutrients [10], the loss of soil fertility [11], and the destruction of agricultural ecosystems, restrict the sustainable development of global agriculture [12,13,14,15], thus requiring urgent attention.

Particularly, microbial treatment, usually using microbial inoculants, is a technology that resolves ecological problems using natural ecological processes. Being non-toxic, environmentally friendly, and sustainable for agricultural development [16,17,18], it has been attracting increasing attention in the last decade and is extensively studied. For example, its role in germination [19,20] and its effect on promoting the growth of plants in greenhouses [21,22] have been investigated. Field experiments with microbial inoculants have also been conducted to explore their agricultural feasibility [23]. Some studies have focused on single strains [24], whereas others, compound treatments, were developed using multiple microbial strains or their combination with components, such as biochar [25], soil amendments with trace elements, and nanomaterials [26]. These studies have shown that microbes from various kingdoms (including fungi and bacteria), phyla (including Acidobacteria, Gemmatimonadetes, Actinobacteria, and Proteobacteria), and genera (such as *Bacillus*, *Pseudomonas*, and *Burkholderia*) promote the growth of diverse plants and exert biocontrol functions against various plant pathogens [27,28,29,30]. However, studies on the growth-promoting or biocontrol mechanisms of functional bacteria remain limited, especially in terms of the interaction between microorganisms and plants [31,32]. Additionally, methods used to explore the mechanism of the plant growth-promoting activity of functional bacteria are mainly related to genomics, and lead to the lack of a systematic understanding of the promoting process.

Metabolomics focuses on the detailed characterization of metabolic phenotypes in a biochemical process [33]. Thus, it is a reliable method to investigate the differences in metabolites produced by plants and their interaction with the microbiome. Untargeted metabolomics has also been used to assess soil quality and microbial function [34]. In particular, the soil rhizosphere metabolite profiles and maize metabolic pathways have been studied using untargeted metabolomics [35]. Additionally, metabolic profiling and quality assessment can be analyzed using untargeted metabolomics not only during growth and development but also postharvest [36]. Accordingly, untargeted metabolomics can be used to observe the changes in overall and specific metabolites, which provides us with insights regarding the plant growth-promoting mechanism of different treatments by the ability to “see” what plants and microbiomes are doing or what they have already done at the molecular level [37].

This study aimed to compare the individual and joint effects of two bacterial strains on maize growth and explore the underlying mechanism of their growth-promoting effects using a metabolomics approach. Two bacterial strains, *Citrobacter amalonaticus* (M16) and *Bacillus safensis* (M44), were screened from maize-planted soil in northeastern China, and their beneficial effects on the germination and growth of maize seedlings were investigated. Furthermore, an untargeted metabolomics approach was utilized to explore the overall changes in specific and concrete metabolites with different treatment groups. Both total and differential metabolites were further profiled to identify the key metabolites associated with plant growth promotion based on the correlation analysis. These findings helped to reveal the metabolic and biochemical mechanism through which the phosphate-solubilizing bacteria (PSB) could promote plant growth.

## 2. Materials and Methods

### 2.1. Screening for PSB

Maize rhizosphere soil samples were collected from a no-tillage maize plantation in Siping, Jilin Province, China and immediately transported to the laboratory under cool conditions. Three sterilized conical flasks (50 mL) were prepared and 3 g soil and 27 mL sterilized 0.85% saline solution (NaCl) were added to each flask. The flasks were shaken at 150 rpm for 20 min at 28 °C and placed without disturbance for 5 min, and serial diluents of 10^−1^ (10^−2^, 10^−3^, 10^−4^, 10^−5^, and 10^−6^) were prepared. A total of 100 µL was transferred and smeared on lysogeny broth (LB) plates (10 g tryptone, 5 g yeast extract, 10 g NaCl, 20 g agar, 1000 mL H2O at pH 7.0–7.2; sterilized at 121 °C for 20 min) [38]. Subsequently, the plates were turned upside down at 28 °C and cultivated for 48 h. Plates with approximately 30–300 colonies were selected, and each colony was transferred into individual plates for cultivation. Each bacterial strain was mixed with 80% glycerin at a ratio of 1:1 and stored at −80 °C. The National Botanical Research Institute’s phosphate (NBRIP) growth medium (10 g glucose, 5 g Ca_3_(PO_4_)_2_, 5 g MgCl_2_, 0.25 g MgSO_4_, 0.2 g KCl, 0.1 g (NH_4_)_2_SO_4_, 1000 mL H_2_O at pH 7.0; sterilized at 115 °C for 30 min) was utilized to initially screen PSB based on the formation of a halo [39]; subsequently, the quantitative test of phosphate-solubilizing ability was further implemented with NBRIP broth. Strains with a high amount of soluble phosphorus were selected for further experiments.

### 2.2. Identification of Bacterial Strains

#### 2.2.1. Sanger Identification

A Bacterial DNA Kit (Omega Bio-tek, Inc., Norcross, GA, USA) was used for DNA extraction according to the manufacturer’s instructions. The full-length 16S rDNA gene was amplified from the bacterial DNA by PCR using barcode 27F (AGAGTTTGATCMTGGCTCAG) and 1492R (CGGTTACCTTGTTACGACTT) primers as described previously [40]. Following PCR amplification, PCR products were subjected to 1% agarose gel electrophoresis and stained with ethidium bromide for 40 min at 100 V. The sequences were aligned using DNAman software (Lynnon Corp., Quebec, Canada) and then compared with the 16S rRNA sequences in the NCBI database [41,42]. Identifications were preliminarily made at a genus/species level based on similarity values of >99%. The top 20 sequences with the highest similarity were selected and then imported into MEGA 7 for the next analysis. A phylogenetic tree was constructed using the neighbor-joining method as implemented in MEGA 7. Sequence alignment was carried out using the ClustalW program based on target 16S rRNA genes. The percentage of replicate trees clustering together in the bootstrap test (500 replicates) was shown next to the branches.

#### 2.2.2. Physiological and Biochemical Identification

The selected strains were cultivated on LB medium plates for Gram’s test. One drop of 5% KOH solution was dripped on a sterilized glass slide, and colonies of each strain were picked using inoculating loops and mixed with the KOH on the glass slides. During the process of mixing, the loops were raised after several seconds to observe whether the wiredrawing phenomena would happen. Strains associated with the wiredrawing phenomenon were identified as Gram-negative bacteria; otherwise, strains without that phenomenon were identified as Gram-positive bacteria. Subsequently, API 20E and API ZYM kits (bioMérieux, Marcy-l’Étoile, France) were used to test the activity of enzymes for Gram-negative and Gram-positive bacteria, respectively [43,44]. Moreover, each strain was tested by API 50CH carbohydrate fermentation analysis as in Mekonnen et al. [45].

### 2.3. Germination of Maize Seeds

Seed germination pouches (PhenoTrait Technology Co., Ltd., Beijing, China) were used as the cultivated carriers instead of soil. A total of 100 mL of LB broth was prepared in 250 mL conical flasks and sterilized at 121 °C for 20 min. The original bacterial solution was transferred to flasks containing sterilized LB broth at a ratio of 1:1000, and the flasks were shaken at 150 rpm for 24 h at 28 °C. M16 and M44 were the pure culture liquids of the two screened bacterial strains, respectively. Com was the culture liquid mixed with 1:1 M16:M44. Provided by the Biological Pesticide Laboratory, Institute of Plant Protection, Jilin Academy of Agricultural Sciences, maize seeds (“Jidan 558“) were washed with distilled water, disinfected in 10% H_2_O_2_ for 15 min, and washed three times with sterilized water [46].

Microbial inoculants cultured for 24 h were adjusted to OD_600_ = 0.8 using sterilized water. Disinfected seeds were immersed in different inoculants for 12 h, and the seeds immersed in sterilized LB broth were labelled as CK. Subsequently, five seeds were placed in each germination pouch according to different treatments (CK, M16 treatment, M44 treatment, and combined treatment of M16 and M44), and each treatment had eight pouches. The corresponding bacterial culture liquid (10 mL) and 10 mL of sterilized water were added to the pouches. In the CK pouches, 10 mL each of sterilized LB broth and sterilized water were added. All pouches were cultivated at 30 °C, and 5 mL sterilized water was added to each pouch daily for 14 days.

### 2.4. Sample Collection

Destructive sampling was performed on the 7th and 14th days by randomly selecting four pouches (replicates) for each treatment. The lengths of the roots and sprouts were measured, and the roots and stems were separated; thus, the underground and aboveground biomasses were weighed. Finally, the pouches were again cut off above the water lines at the bottom, and the culture liquid was collected in 50 mL sterilized Corning tubes and stored at −80 °C.

### 2.5. Metabolomics Analysis and Data Processing

#### 2.5.1. Metabolite Extraction

The culture liquid samples (100 µL) were mixed with cold 80% methyl alcohol in centrifuge tubes, vortexed, placed on ice without disturbance for 5 min, and centrifuged at 15,000× *g* at 4 °C for 20 min. LC-MS grade water was used to dilute the supernatant into a concentration containing 53% methanol. Subsequently, the samples were transferred into new tubes and centrifuged under the same conditions described previously in this section. Finally, the supernatant was injected into the LC-MS/MS system for further analysis [47,48].

#### 2.5.2. UHPLC-MS/MS Analysis

UHPLC-MS/MS analyses were performed using a Vanquish UHPLC system (ThermoFisher, Bremen, Germany) coupled with an Orbitrap Q ExactiveTM HF mass spectrometer (Thermo Fisher) at Novogene Co., Ltd. (Beijing, China) using the following parameters: spray voltage, 3.2 kV; capillary temperature, 320 °C; sheath gas velocity, 40 arb; and auxiliary gas velocity, 10 arb. For the positive-ionization mode, the mobile phase A was 0.1% formic acid in water, whereas the mobile phase B was methanol. For the negative-ionization mode, mobile phase A was 5 mM ammonium acetate at pH = 9.0, whereas the mobile phase B was methanol.

#### 2.5.3. Data Processing and Metabolite Identification

The raw data from UHPLC-MS/MS analyses were processed using Compound Discoverer 3.1 (Thermo Fisher) for peak alignment, peak picking, and the quantitation of each metabolite. Processed data were normalized according to the total spectral intensity, and the normalized data were used to predict the molecular formula of each metabolite based on molecular ion peaks, fragment ions, and additive ions. Meanwhile, the results of the peaks were matched against the mzCloud, mzVault, and MassList databases to obtain qualitative and relative quantitative results.

#### 2.5.4. Data Analysis

All detected metabolites were matched against the KEGG (https://www.genome.jp/kegg/pathway.html, accessed on 30 January 2021) and LIPIDMaps databases (http://www.lipidmaps.org/, accessed on 30 January 2021). PLS-DA was initially used to determine the variation among different treatments and obtain the VIP value. Metabolites with VIP > 1, *p* < 0.05, and fold-change (FC) ≥ 2 or FC ≤ 0.5 were considered as differential metabolites, which were used for further analyses.

### 2.6. Statistical Analysis

Raw data were preprocessed using Microsoft Excel 2016, and analysis of variance (ANOVA) was implemented using IBM SPSS Statistics software (version 25.0; Chicago, IL, USA). Comparisons between single microbial treatments and CK were analyzed using a *t*-test, whereas ANOVA was used for multiple comparisons. The significance of correlations between differential metabolites and KEGG enrichment analyses were calculated using the *p*-values of corresponding R packages (ggcorrplot, clusterProfile, and vegan). Data are presented as the mean ± standard deviation.

## 3. Results

### 3.1. Screening PSB

From the strains that formed a halo on NBRIP medium plates, five were selected for quantitative experiments of phosphate-solubilizing ability. The quantitative results (Table 1) show that the amounts of soluble phosphorus released by M10, M16, M44, M101, and M262 strains were significantly higher if compared to control (CK). Among them, M16 and M44 solubilized approximately 4−6-fold more phosphate than the other three strains. Additionally, there was a significant difference in soluble phosphorus between M16 and M44.

Based on the quantitative results of phosphate-solubilizing ability, M16 and M44 were selected for further identification. From the sequencing results of 16S rRNA (Figure S1), M16 was initially identified as *Citrobacter* spp. (Figure S1A), whereas M44 was initially identified as *Bacillus* spp. (Figure S1B). Subsequently, the results of Gram’s test showed that M16 was a Gram-negative bacterium, whereas M44 was a Gram-positive bacterium (Table S1). Additionally, the results of enzyme activities indicated that M16 had positive reactions for β–galactosidase, arginine dihydrolase, ornithine decarboxylase, indole production, and acetoin production (Table S2). M44 had positive reactions for alkaline phosphatase, esterase (C4), esterase lipase (C8), trypsin, acid phosphatase, naphthol-AS-BI-phosphohydrolase, and β-glucosidase (Table S3). Furthermore, tests for the utilization of carbon sources showed that M16 could produce acids using 22 of 49 carbon sources (tested with API 50CH acid kits), such as glycerol, D-ribose, lactose, D-galactose, D-glucose, and D-fructose (Table S4), whereas M44 could use 25 of 49 carbon sources, many of which were similar to those utilized by M16. Interestingly, M44 could use sucrose to produce acid, whereas M16 could not (Table S5). Other differences in carbon source utilization between M16 and M44 were mainly focused on D-arabinose, sorbose, rhamnose, and melibiose, among others. Combining the results of 16S rRNA sequencing identification and physiological and biochemical identification, M16 was identified as a *Citrobacter amalonaticus* (GenBank number: MW362493), whereas M44 was identified as a *Bacillus safensis* (GenBank number: MW362494).

### 3.2. Bacterial Growth Promotion of Maize Seedlings

**Figure 1:**
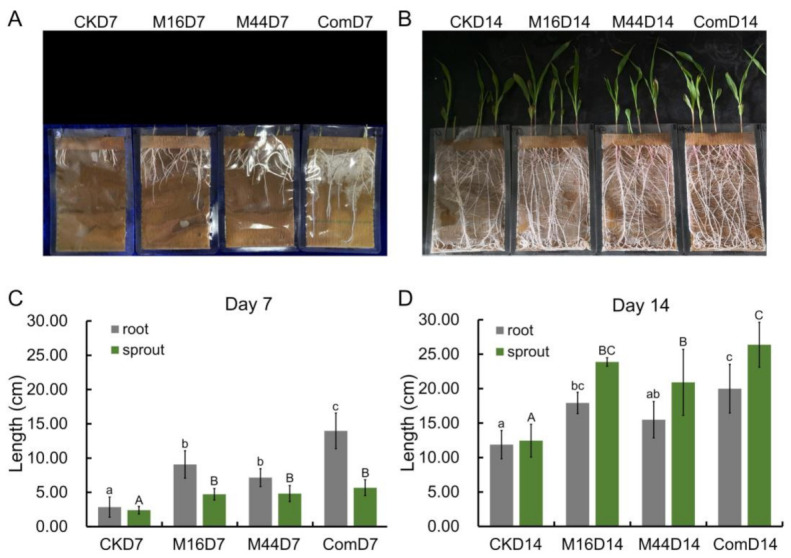
Maize germination for 14 days following microbial treatment. (**A**) Photograph showing root length at day 7 (D7). (**B**) Photograph showing root and sprout length at day 14 (D14). Bar graphs showing the root and sprout lengths at D7 (**C**) and D14 (**D**). CK: control; M16: M16 treatment; M44: M44 treatment; Com: combination of M16 and M44. Columns with the same lower (represent the comparisons of roots) and upper-case (represent the comparisons of sprouts) letters are not significantly different at the 5% level by DMRT, respectively.

To investigate the growth-promoting abilities of the two strains, seed germination pouches were utilized in laboratory experiments of maize from the germination to seedling stage. From the apparent growth of maize seedlings in seed germination pouches, obvious differences were observed between the microbial treatments and the CK. At day 7 (D7), seedlings subjected to microbial treatments developed longer roots, and at day 14 (D14), they were stronger with more lateral roots than those in the CK group. The seeds subjected to microbial treatments sprouted earlier and faster at D7 and had higher and sturdier seedlings at D14 than CK (Figure 1A,B).

Particularly, at D7, the root lengths following treatment with M16, M44, and Com were 9.08 ± 2.01, 7.15 ± 1.30, and 13.98 ± 2.61 cm, respectively. These values were all significantly higher than those of CK (2.85 ± 1.47 cm; *p* < 0.05). Seedlings treated with Com had the longest roots among the three microbial treatments (*p* < 0.05), whereas there was no significant difference in root length between M16 and M44 treatments. Similar observations were found in terms of sprout length; all three microbial treatments (4.73 ± 0.84, 4.83 ± 1.17, and 5.68 ± 1.16 cm, respectively) resulted in significantly longer sprouts than the CK treatment (2.40 ± 0.55 cm; *p* < 0.05). However, there was no significant difference in sprout length among all microbial treatments (Figure 1C). At D14, all three microbial treatments had significantly (*p* < 0.05) longer roots (M16, 17.93 ± 1.55 cm; Com, 20.00 ± 3.53 cm) and sprouts (M16, 23.88 ± 0.63 cm; M44, 20.93 ± 4.79 cm; Com, 26.38 ± 3.25 cm) than those of the CK group (root, 11.88 ± 2.06 cm; sprout, 12.45 ± 2.38 cm), except for the root length of M44 vs. CK. In terms of root and sprout lengths, Com was significantly different from M44, whereas there were no significant differences in the comparisons of Com vs. M16 and M16 vs. M44 (Figure 1D).

The biomass of all treatments was measured at the fourteenth day (D14). Based on biomass, the root weights of seedlings treated with M16, M44, and Com were significantly (*p* < 0.05) larger than those in the CK group. In terms of the mass of sprouts, those subjected to microbial treatments were significantly (*p* < 0.05) heavier than those in the CK group. Furthermore, among the microbial treatments, there were significant differences between Com and M44, whereas in terms of root mass, there was no significant difference in the Com vs. M16 and M16 vs. M44 comparisons, which was in accordance with the results of root length at D14. Additionally, M16 and Com treatments resulted in significantly (*p* < 0.05) heavier sprout weight than M44 treatment, whereas there was no significant difference between M16 and Com (Table 2).

### 3.3. Overview of Metabolomic Annotation

To understand the beneficial effects of microbial inoculants (including M16, M44, and Com) on the germination and growth of maize at the molecular level, the untargeted metabolomic method, which was based on UHPLC-MS, was used to explore all detectable metabolites produced during development from maize seeds to seedlings. Overall, we annotated 1142 compounds in the positive-ionization mode and 643 compounds in the negative-ionization mode. The most clustered annotations of the KEGG pathway included metabolism, with 298/324 metabolites in the positive-ionization mode and 263/273 metabolites in the negative-ionization mode. For secondary metabolism annotations, global and overview maps, amino acid metabolism, metabolism of cofactors and vitamins, and lipid metabolism were the distinct categories in the positive-ionization mode, and global and overview maps, amino acid metabolism, lipid metabolism, and metabolism of cofactors and vitamins were identified in the negative-ionization mode (Figure S2A,B). Additionally, the main categories based on Lipidmap annotation included fatty acids (FAs), glycerophospholipids (GPs), and sterol lipids (STs) in both the positive- and negative-ionization modes, with metabolite numbers of 46, 27, and 22 and 52, 42, and 14, respectively. Nevertheless, the secondary annotations of FA, GP, and ST were different between the two ionization modes. For example, eicosanoids (FA03), glycerophosphoethanolamines (GP02), and steroids (ST02) were the top FA, GP, and ST, respectively, in the positive-ionization mode; meanwhile, fatty acids and conjugates (FA01), glycerophosphoglycerols (GP04), and steroid conjugates (ST05) were those in the negative-ionization mode (Figure S2C,D). The amounts of metabolites associated with plant growth (including root development and sprout growth) and bacterial cultivation were also detected by different ionization modes (positive and negative).

### 3.4. Metabolomic Composition and Structural Variation

**Figure 2:**
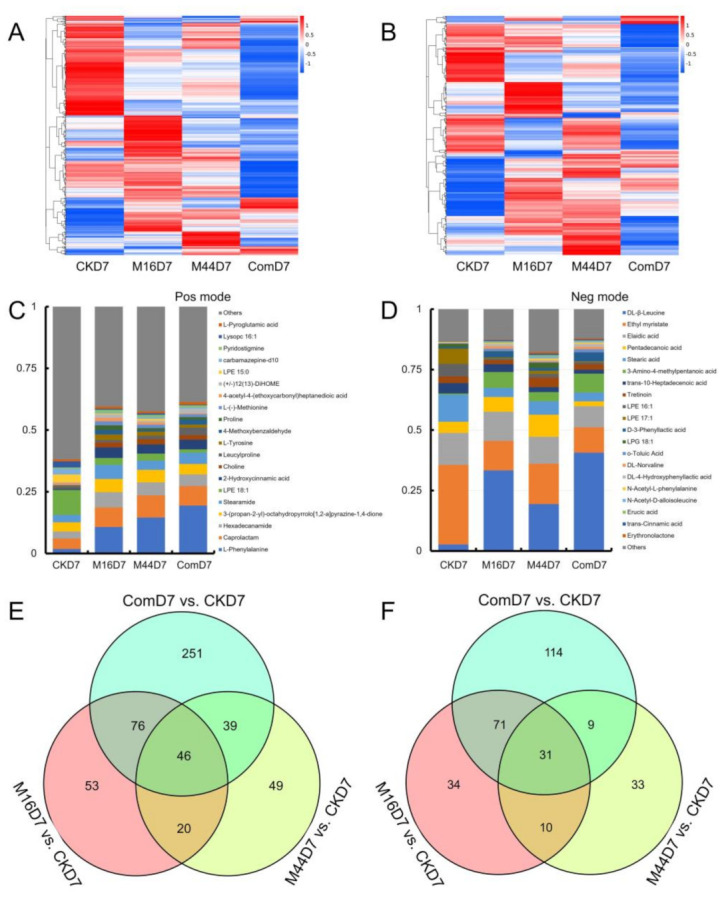
Metabolomic composition and structural variation with different treatments. (**A**) Heatmap of all metabolites detected from different treatments in the positive-ionization mode. (**B**) Heatmap of all metabolites detected from different treatments in the negative-ionization mode. (**C**) Relative abundance of top 20 metabolites from all treatments in the positive-ionization mode. (**D**) Relative abundance of top 20 metabolites from all treatments in the negative-ionization mode. (**E**) Venn diagram showing the differential metabolites among group comparisons in the positive-ionization mode. (**F**) Venn diagram showing the differential metabolites among group comparisons in the negative-ionization mode. CK: control; M16: M16 treatment; M44: M44 treatment; Com: combination of M16 and M44; D7: day 7.

Since the general situation of the metabolomic annotation was scanned, the metabolomic composition associated with different treatments and the variation between them were studied. Hence, heatmaps based on normalized statistics of metabolites in different treatment groups were first implemented to show the similarities and dissimilarities among all treatment groups according to their metabolic profiles. Heatmaps showed clear variations among different treatments in both positive- and negative-ionization modes. In particular, the differentiation between microbial treatments and CK was wider than that among all microbial treatments (Figure 2A,B). Interestingly, there was a significant change in the composition of metabolites when M16 and M44 were used in the combined formulation (Com), whereas the gap in metabolomic composition between M16 and M44 was not that large (Figure 2A,B). Similar variations in metabolomic composition were found with different treatments at D14, although the degree of differentiation among M16, M44, and Com was slightly reduced compared with that at D7 (Figure S3A,B).

To further determine the detailed differences in metabolomic composition among the different treatments, the top 20 metabolites of all treatments were computed and selected. These were pooled from each treatment and presented as histograms (Figure 2C,D and Figure S3C,D). The top 20 metabolites could quantitatively distinguish the different treatments, which indicated that this approach did differentiate between the three microbial treatments and CK in both ionization modes. The quantitative compositions of the top 20 metabolites in M16, M44, and Com were similar in the positive-ionization mode, with top-ranking metabolites of l-phenylalanine, caprolactam, and hexadecanamide. However, only the first three metabolites were consistent in the negative-ionization mode (Figure 2C,D). Particularly, in M16, 3-amino-4-methylpentanoic acid and pentadecanoic acid, respectively, ranked fourth and fifth, whereas in M44, pentadecanoic acid and stearic acid, and in Com, 3-amino-4-methylpentanoic acid and stearic acid held these positions. The metabolomic compositions of the top 20 metabolites were similar at D7 and D14; the variation between the microbial treatments and CK decreased compared with that at D7 (Figure S3C,D).

To identify the key metabolites that dominated the growth-promoting effect of microbial stimulators, significantly (*p* < 0.05) different metabolites between M16, M44, or Com and CK were further explored. Venn diagrams were used to reflect the structural variation of significant differences in differential metabolites between different comparative groups (M16 vs. CK, M44 vs. CK, and Com vs. CK) based on the overlap of all metabolites (Figure 2E,F and Figure S3E,F). At D7, M16, M44, and Com had 53, 49, and 251 unique differential metabolites (vs. CK) in the positive-ionization mode and 34, 33, and 114, respectively, in the negative-ionization. There were 46 and 31 shared differential metabolites among M16, M44, and Com, respectively, in both positive- and negative-ionization modes. M16 and Com had the most shared differential metabolites, whereas M16 and M44 had the fewest shared metabolites (Figure 2E,F). At D14, the number of shared differential metabolites decreased in all comparisons. There were 31, 25, and 35 unique differential metabolites of M16, M44, and Com in the positive-ionization mode, and 21, 23, and 10 in the negative-ionization mode, respectively. Interestingly, M44 and Com had the greatest number of shared metabolites in both modes, which was divergent from that at D7 (Figure S3E,F).

### 3.5. Functional Analysis of Differential Metabolites and Integration of Metabolomic Community

We then explored the correlations among the top 20 differential metabolites based on different treatment comparisons using heatmaps. Generally, the main differential metabolite families in the three microbial treatments were amino acids, molecular organic acids, and acylated amino acids (Figure S4). Based on the combined results of all top 20 differential metabolites (including positive and negative modes), eight metabolites were shared by the three microbial treatments concurrently. Those upregulated include the amino acid dl-norvaline; molecular organic acids, 2-hydroxycinnamic acid, 3-amino-4-methylpentanoic acid, and trans-cinnamic acid; and acylated amino acids, N-acetyl-d-alloisoleucine and N-acetyl-l-methionine. In contrast, the remaining two metabolites, LPE 19:1 and 2-methoxy-5-(1H-1,2,4-triazol-5-yl)-4-(trifluoromethyl) pyridine, were downregulated. Meanwhile, the upregulated metabolites shared by M16 and M44 mainly included l-tyrosine, d-phenylalanine, proline, caprolactam, choline, N-benzyl-N-isopropyl-N′-[4-(trifluoromethoxy) phenyl] urea, and 2-(acetylamino)-4-(methylthio) butanoic acid. Only one metabolite shared by M44 and Com (l-phenylalanine) was upregulated. M16 and Com shared 15 metabolites, of which dl-β-leucine, α-linolenic acid, pilocarpine, and 4-methoxybenzaldehyde were upregulated, whereas carbamazepine-d10, 1,4-dihydroxyheptadec-16-en-2-yl acetate, 1-(4-benzylpiperazine)-2-(pyridin-2-ylamino) propan-1-one, adenosine, LPE 17:1, α, α-trehalose, and four different formations of LPG (LPG 16:0; 16:1; 18:1; 19:1) were downregulated. In addition, 9, 24, and 15 unique metabolites were found in M16, M44, and Com, respectively, most of which were molecular organic acids and amino acids. Interestingly, the top 20 amino acids and molecular organic acids were positively correlated with each other and more likely to promote plant growth. Substances such as LPG and DiHOME were negatively related to these beneficial metabolites. Among them, the series of LPG (16:0, 16:1, 17:1, 18:1, and 19:1) showed distinct contrasting trends compared with those of other beneficial metabolites.

**Figure 3:**
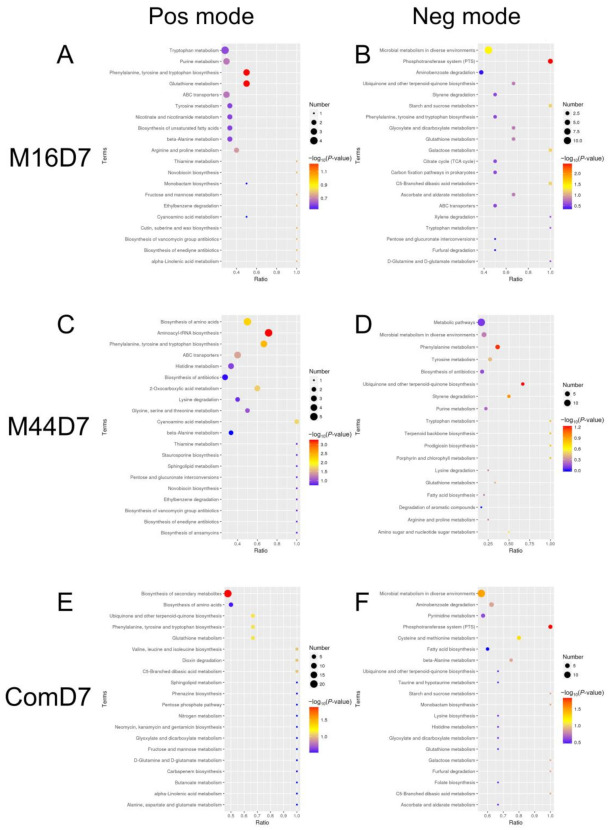
Bubble diagram of KEGG pathway enrichment analysis. (**A**) M16D7 in positive mode. (**B**) M16D7 in negative mode. (**C**) M44D7 in positive mode. (**D**) M44D7 in negative mode. (**E**) ComD7 in positive mode. (**F**) ComD7 in negative mode. CK: control; M16: M16 treatment; M44: M44 treatment; Com: combination of M16 and M44; D7: day 7.

To focus on the functional variation caused by the differential metabolites, KEGG pathway enrichment analysis was performed for each microbial treatment against CK (Figure 3). The top 20 pathways were identified, and five were shared by all three microbial treatments. Most were associated with the biosynthesis and/or metabolism of amino acids and proteins, including the biosynthesis of phenylalanine, tyrosine, and tryptophan; the metabolism of beta-alanine; and the metabolism of glutathione. Microbial metabolism in diverse environments and biosynthesis of ubiquinone and other terpenoid-quinones were also shared by the three treatments. Additionally, M16 and M44 had 13 shared pathways with more different kinds of metabolic pathways. Specifically, the metabolism of arginine and proline; metabolism of tryptophan; and metabolism of tyrosine were the main pathways involved in the metabolism of amino acids; ABC transporters were associated with the metabolism of protein, biosynthesis of different antibiotics (including novobiocin; vancomycin group; and enediyne), and the degradation and metabolism of organic molecules (degradation of ethylbenzene; degradation of styrene; metabolism of thiamine; metabolism of purine; and metabolism of cyanoamino acid), and interconversions between pentose and glucuronate were among the shared pathways. Furthermore, M16 and Com had 12 shared pathways, which were mainly related to the metabolism of carbohydrates, molecular organic acids, and amino acids, accounting for 75% of the shared pathways. The remaining three included the phosphotransferase system (PTS); degradation of aminobenzoate; and the biosynthesis of monobactam. Nevertheless, M44 and Com had only four shared pathways, including the biosynthesis of amino acids; metabolism of histidine; metabolism of sphingolipid; and biosynthesis of fatty acid. In addition, 6, 16, and 15 unique pathways were enriched in M16, M44, and Com, respectively, based on the KEGG database. The unique pathways enriched in M16 were the citrate cycle (TCA cycle), carbon fixation, and those associated with plant growth regulators, such as nicotinate, nicotinamide, and unsaturated fatty acids. When it came to unique pathways in M44, the metabolism of different amino acids and biosynthesis of aminoacyl-tRNA, antibiotics, staurosporine, and terpenoid backbone were dominant, whereas in Com, the unique pathways were mainly focused on the metabolism of several amino acids and antibiotics (including carbapenem, phenazine, neomycin, kanamycin, and gentamicin). Meanwhile, the pathways related to the metabolism of nitrogen and several plant growth regulators (such as pyrimidine, folate, taurine, and hypotaurine) were uniquely enriched in Com as well.

**Figure 4:**
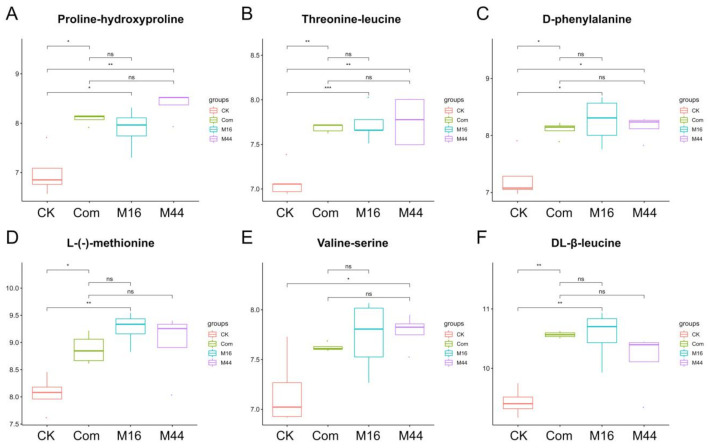
Boxplots of differential amino acids, proline-hydroxyproline (**A**), threonine-leucine (**B**), D-phenylalanine (**C**), L-(-)-methionine (**D**), valine-serine (**E**), and DL-β-leucine (**F**), based on a *t*-test. CK: control; M16: M16 treatment; M44: M44 treatment; Com: combination of M16 and M44. Asterisks between different treatments represent significance (* means 0.01 < *p* < 0.05; ** means 0.001 < *p* < 0.01; *** means *p* < 0.001).

**Figure 5:**
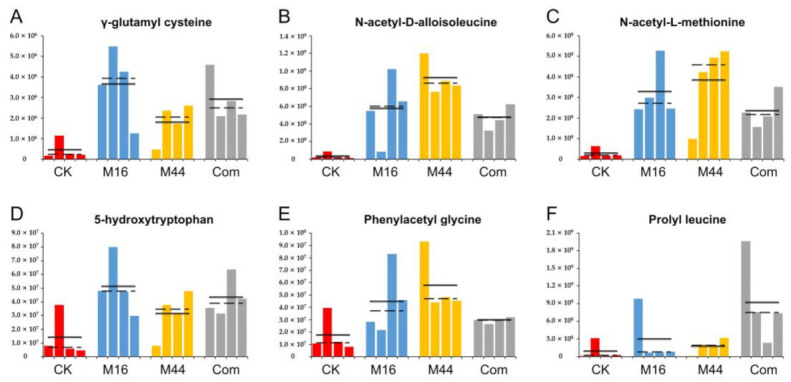
Histograms of differential amino acid derivatives, γ-glutamyl cysteine (**A**), N-acetyl-D-alloisoleucine (**B**), N-acetyl-L-methionine (**C**), 5-hydroxytryptophan (**D**), phenylacetyl glycine (**E**), and prolyl leucine (**F**), based on quantitative statistics. CK: control; M16: M16 treatment; M44: M44 treatment; Com: combination of M16 and M44. Solid and dashed lines represent the means and medians of the contents of metabolites in CK, M16, M44, and Com, respectively.

**Figure 6:**
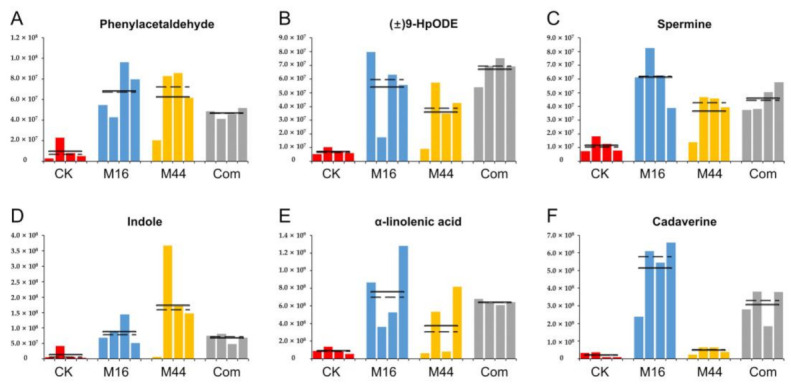
Histograms of plant growth-regulating molecules, phenylacetaldehyde (**A**), (±)9-HpODE (**B**), spermine (**C**), indole (**D**), α-linolenic acid (**E**), and cadaverine (**F**), based on quantitative statistics. CK: control; M16: M16 treatment; M44: M44 treatment; Com: combination of M16 and M44. Solid and dashed lines represent the means and medians of the contents of metabolites in CK, M16, M44, and Com, respectively.

Since the enriched KEGG pathways had been investigated, to deeply uncover the key metabolites that participated in pathways closely related to plant growth-promoting functions, biomarkers of metabolites from different treatments were further explored. Based on the results of correlations between differential metabolites and plant growth, including a pairwise correlation (Table S6) and mantel test (Table S7), many metabolites were found to be significantly (*p* < 0.05) correlated with maize growth, from which three categories were summarized as amino acids, amino acid derivatives, and other plant growth-regulating molecules. Among them, only six metabolites from each category were exhibited (Figure 4, Figure 5 and Figure 6). Firstly, the amino acids were the focus due to their active participation in various processes of plant growth and development. According to the statistical results based on a *t*-test, proline-hydroxyproline, threonine-leucine, and D-phenylalanine were much more prevalent in M16, M44, and Com (vs. CK), and these were defined as differential amino acids shared by all three microbial treatments (Figure 4A–C). Furthermore, there was a significantly (*p* < 0.05) higher concentration of L-(-)-methionine and DL-β-leucine in both M16 and Com (vs. CK), whereas no significant differences were found between M44 and CK (Figure 4D,F). Additionally, more (*p* < 0.05) valine-serine was found only in M44 (vs. CK), whereas there were no significant differences among M16, Com, and CK (Figure 4E).

In addition to amino acids, amino acid derivatives were investigated based on the quantitative results through ANOVA (Figure 5). γ-glutamyl cysteine, N-acetyl-D-alloisoleucine, and N-acetyl-L-methionine were found as shared differential (*p* < 0.05) metabolites by all three microbial treatments (vs. CK); among them, M44 had significantly (*p* < 0.05) larger amounts of N-acetyl-D-alloisoleucine than M16 and Com, whereas the three microbial treatments had no significant differences in γ-glutamyl cysteine and N-acetyl-L-methionine (Figure 5A–C). Interestingly, the contents of 5-hydroxytryptophan, phenylacetyl glycine, and prolyl leucine were significantly (*p* < 0.05) enhanced by M16, M44, and Com (vs. CK), respectively (Figure 5D–F). This indicated that these metabolites were separately unique to the three microbial treatments.

Furthermore, apart from amino acids and their derivatives, other plant growth-regulating molecules such as polyamine, auxin, and molecular organic acid were explored to improve the understanding of the metabolomic mechanism of the plant growth-promoting effects of microbial inoculants (Figure 6). The histograms showed that the production of phenylacetaldehyde, (±)9-HpODE, spermine, and indole were improved by all three microbial treatments (vs. CK). Among them, M16 and Com had more spermine and (±)9-HpODE than M44 (*p* < 0.05), respectively, whereas the three microbial treatments had no significant differences in phenylacetaldehyde and indole (Figure 6A–D). Moreover, the contents of α-linolenic acid (E), and cadaverine were significantly (*p* < 0.05) increased by M16 and Com (vs. CK), whereas the differences between M44 and CK in those two metabolites did not reach statistical significance (Figure 6E,F). Interestingly, for cadaverine, the content was much higher with M16 than with Com (*p* < 0.05), and it was also much higher with Com than with M44 (*p* < 0.05).

## 4. Discussion

To address agricultural problems such as overuse of chemical fertilizer, reduction in yield, and decrease in quality in crops, an environmentally friendly, harmless, and sustainable solution is urgently needed. The application of microbial inoculants, which was found to promote the growth and yield of crops [49], enhance the induced systemic resistance (ISR) of plants [50], and maintain the stability of farmland ecosystems [51], is a feasible strategy against current agricultural problems. To better develop and apply this strategy, it is necessary to understand the mechanisms underlying the plant growth-promoting effects of microbial treatments. Herein, two PSB were selected, and their performance in promoting plant growth were investigated through a seed germination pouch method, and significant improvements of maize roots and sprouts (lengths and weights) were found with the three microbial treatments (M16, M44, and Com). Among them, Com performed the best. Furthermore, the total and differential metabolites were identified and their variations among different treatments were analyzed. Based on correlation analyses between differential metabolites and plant growth indexes, 48 metabolites, which were significantly correlated with plant growth, were identified and summarized in three categories (amino acids, amino acid derivatives, and other plant growth-regulating molecules). Consistent with their performances in promoting plant growth, Com had more kinds of these key metabolites than M16 and M44, which indicated the promoting effects of microbial inoculants were enhanced by the combined application of M16 and M44.

PSB constitute an important microbial taxon that converts insoluble phosphorus into forms available to plants. Based on their ability to secrete organic acids, acid phosphatase, and alkaline phosphatase, PSB lower the pH of the microzone in the rhizosphere and release phosphorus from insoluble rock phosphate (inorganic form) and insoluble organophosphorus (organic form), thereby improving the absorption of phosphorus by plants [52]. Interestingly, an increasing number of studies has shown that in addition to solubilizing phosphate, PSB possess other traits that promote plant growth. For example, some can produce indole-3-acetic acid (IAA), a well-known plant growth regulator [53]. Some can secrete organic molecules from which plants gain resources for their growth directly [54]. For example, plants could use organic carbon as a carbon source [55]; moreover, they could absorb urea derivatives such as N-benzyl-N-isopropyl-N′-[4-(trifluoromethoxy)phenyl]urea to supplement their nitrogen store [56]. Additionally, as signal molecules, some molecular organics (such as polyamines, phenolic acid, linolenic acid, etc.) play important roles in plant development [57]. Furthermore, the absorption of phosphorus by plants can affect that of nitrogen. Meanwhile, the use-efficiency of other nutrient elements has been associated with the absorption of phosphorus [58]. Moreover, PSB can enhance the ISR of plants against pathogens and environmental stresses. Therefore, PSB can promote plant growth in maize and address current agricultural challenges.

Among all strains in this study, the soluble phosphorus at the seventh day released by M16 and M44 reached 444.88 ± 13.31 and 577.54 ± 33.99 mg L^−1^, which was significantly higher than the other three strains. According to recent research, the amount of soluble phosphorus treated by PSB mainly ranged from 100 to 500 mg L^−1^ [59]; the lowest number could be about 20–30 mg L^−1^ [60]. Thus, M16 and M44 are 1.5–2.0 times more than the average. Generally, it is acceptable to further study PSB when the amount of soluble phosphorus is more than 100 mg L^−1^ [61], and PSB whose phosphate-solubility was more than 300 mg L^−1^ could be regarded as highly efficient PSB [62,63]. Furthermore, the plant growth-promoting abilities of M16, M44, and Com were explored; at D7, M16, M44, and Com increased the root and sprout lengths by 219 and 97, 151 and 101, and 391 and 137%, respectively, relative to those of CK, whereas at D14, these increases were 51 and 92, 30 and 68, and 68 and 112%, respectively. While Viruel et al. found that the plant height and shoot weight were improved by 45 and 40%, respectively, at D30 [64]. The percentages were larger at D7 than at D14 and D30 because plants grow much faster in the early stage of germination [65]. Therefore, the variation in the growth of maize seedlings in the early stage was greater than in the later stage. The combination of M16 and M44 enhanced the growth-promoting effect, especially compared with that of M44. This finding was consistent with other studies on multiple inoculants’ treatments [66], and the tendency of a synergistic effect to increase maize growth could be enhanced when more strains were involved in the mixed bacterial inoculants [67].

After demonstrating improvements in the lengths and biomasses of roots and sprouts, we investigated the metabolomic profiles to explore the mechanism underlying growth promotion. Microbial treatments vastly changed the composition of total metabolites. From all differential (*p* < 0.05) metabolites (vs. CK), whose productions were enhanced by M16, M44, and Com (commonly or uniquely), 48 metabolites were found to be significantly correlated with plant growth (Tables S6 and S7). From them, three categories were summarized as amino acids, amino acid derivatives, and other plant growth-regulating molecules. Amino acids have important roles in various physiological processes in plants (such as the biosynthesis of proteins, regulating the production of enzymes, and inducing system resistance of plants against stresses [68]) with respect to plant growth and development during its entire life [69]. Popko et al. studied the effect of bio-stimulants based on amino acids on winter wheat, which indicated that amino acids could help increase the yield and improve the quality of crops [70]. Most of the amino acids enhanced by microbial treatments in this study had been reported to benefit plant growth. For example, proline (proline-hydroxyproline) could promote plant growth and improve the apparent characteristics [71], leucine was regarded as a stimulator of plant growth, regulating the photosynthesis of crops [72], valine and serine are the precursors of auxin, and methionine, phenylalanine, and threonine were found to be associated with germination [73]. In addition, phenylalanine was reported to be a precursor of anthocyanin, and could promote the biosynthesis of lignin [74]. In addition to these amino acids, various amino acid derivatives were also increased by microbial treatments. Among these derivatives (Tables S6 and S7), the dominant molecules were acylated amino acids, such as γ-glutamyl cysteine [75], N-acetyl-D-alloisoleucine [76], N-acetyl-L-methionine [77], etc., which were proven to be protective compounds for plants; precursors of polyamines and plant endogenous hormone; and had abilities of promoting plant growth. It is believed that the acylation of amino acids could protect the structure of these amino acids and make them more specific in implementing their functions. Therefore, the greater the content of acylated amino acids demonstrated, the more active amino acids involved in processes of plant growth.

Furthermore, the contents of other plant growth-regulating molecules, such as polyamines, aldehydes, and molecular organic acids, were significantly (*p* < 0.05) increased by microbial treatments as well. Previous research indicated that spermine modulated several biological processes in plants during their development and growth, including root growth, cell division, and gene expression [78]. Cadaverine enhanced the plant resistance against acid and drought stress [79]; meanwhile, it was verified to modulate the root system and promote shoot development based on several aspects [80]. (±)9-HpODE is an inhibitor of lipoxygenase, which oxygenates the double bonds of linoleic acid, linolenic acid, and arachidonic acid to form peroxides, resulting in the destruction of molecular organic acids. According to the research implemented by Kokkiligadda et al., molecular organic acids such as α-linolenic acid and arachidonic acid were signal molecules that participated in modulating multiple processes of plant growth and development [81]. Additionally, indole was an important precursor of IAA, which was well known for its function in promoting plant growth. Nevertheless, few studies on the direct effect of phenylacetaldehyde on plant growth have been performed. Due to its volatility, phenylacetaldehyde is easily converted into phenylethanol and phenylacetic acid, which have increased bioactivities in the regulation of plant growth.

From 48 key metabolites significantly associated with plant growth promotion, some were commonly shared by all three microbial treatments, some were just shared by two of them (shared by M16 and M44, M16 and Com, or M44 and Com), and others were uniquely possessed by one of them (only possessed by M16, M44, or Com), which could be regarded as metabolic biomarkers of plant growth-promoting bacteria. M16 and Com had more shared and unique metabolic biomarkers than M44, which was consistent with their performances in promoting plant growth. It is worth mentioning that the discovery of biomarkers could potentially be used to either estimate the microbial ability to promote plant growth, or monitor interactions, which are associated with plant growth and development, between plants and microorganisms in the rhizosphere.

## 5. Conclusions

In this study, the combination of *Citrobacter amalonaticus* (M16) and *Bacillus safensis* (M44) had the best growth-promoting effects on maize seedlings among the three microbial treatments, with M16 performing better than M44. The variation in compositions of total and differential metabolites among different treatments was highly consistent with their performance in promoting maize growth. A total of 48 key differential metabolites were found to be significantly correlated with maize plant growth, from which three main categories, namely amino acids, amino acid derivatives, and other plant growth-regulating molecules, were summarized. The profiling of these three categories of differential metabolites indicated that the microbial treatments might promote plant growth through modulating the production of those key metabolites, which revealed the metabolic mechanism of the screened PSB in plant growth promotion. This study provides a theoretical basis for studies investigating the plant growth-promoting mechanism of PSB and their application in sustainable agriculture. The application of microbial inoculants and the metabolomics of interactions among plants, microorganisms, and the soil environment should be further researched.

## Figures and Tables

**Table 1:** The amount of soluble phosphorus at day 7 cultivated by different strains.

Strains	Soluble Phosphorus/mg L^−1^
CK	27.03 ± 3.62 ^a^
M10	109.27 ± 8.95 ^b^
M16	444.88 ± 13.31 ^c^
M44	577.54 ± 33.99 ^d^
M101	101.93 ± 35.32 ^b^
M262	99.37 ± 2.44 ^b^

CK: NBRIP medium was inoculated with sterilized LB, and cultivated for seven days; M10, M16, M44, M101, and M262: NBRIP media were inoculated with M10, M16, M44, M101, and M262, respectively, and they were cultivated for seven days; M10, M16, M44, M101, and M262 were cultivated in LB medium before being inoculated onto NBRIP. Means followed by the same letter are not significantly different at the 5% level by Duncan’s multiple range test (DMRT).

**Table 2:** Mass of roots and sprouts at D14 after different treatments.

Treatment	Mass of Root (g)	Mass of Sprout (g)
CK	16.60 ± 1.34 ^a^	7.46 ± 0.52 ^a^
M16	20.78 ± 1.41 ^bc^	9.95 ± 0.43 ^c^
M44	20.39 ± 1.37 ^b^	9.07 ± 0.25 ^b^
Com	22.42 ± 0.58 ^c^	10.16 ± 0.41 ^c^

CK: control; M16: M16 treatment; M44: M44 treatment; Com: combination of M16 and M44. Means in the same column followed by the same letter are not significantly different at the 5% level by DMRT.

## Data Availability

The data presented in this study are available in this article and the supplementary materials.

## Supplementary Materials

The following are available online at https://www.mdpi.com/article/10.3390/plants10061071/s1, Figure S1: Phylogenetic trees based on 16S rRNA sequencing alignments using the neighbor-joining method, Figure S2: Annotation of metabolites following microbial treatment, Figure S3: Metabolomic composition and structural variation of different treatments (D14), Figure S4: Correlations among the top 20 significantly differential metabolites in different comparative groups (M16 vs. CK, M44 vs. CK, Com vs. CK) in two ionization modes. Table S1: Results of Gram’s test, Table S2: Results of enzyme activities of M16, Table S3: Results of enzyme activities of M44, Table S4: Utilization of carbon sources by M16 to produce acids, Table S5: Utilization of carbon sources by M44 to produce acids, Table S6: Pairwise correlations between differential metabolites and plant growth, Table S7: The correlation between differential metabolites and plant growth using the Mantel Test.
