# Supplementary material for: Profiling of Plant Growth-Promoting Metabolites by Phosphate-Solubilizing Bacteria in Maize Rhizosphere
Source: Plants (Basel). 2021 May 27;10(6):1071. doi: 10.3390/plants10061071 (PMC8229199; doi:10.3390/plants10061071)
Supplement: Supplementary file 1 [file plants-10-01071-s001.zip › plants-1169264-supplementary/Supplementary Materials/Figure legends.pdf]

## Figure legend

Figure S1. Phylogenetic trees based on 16S rRNA sequencing alignments using the neighbor-joining method. (A) Phylogenetic tree of M16, taking *Mycobacterium tuberculosis* H37Rv (NR 102810.2) as its out branch. (B) Phylogenetic tree of M44, taking *ALactobacillus delbrueckii subsp. lactis* DSM 20072 (Y050173.1) as its out branch.

Figure S2. Annotation of metabolites following microbial treatment. (A) KEGG pathway in the positive-ionization mode. (B) KEGG pathway annotation in the negative-ionization mode. (C) Lipidmaps in the positive-ionization mode. (D) Lipidmaps annotation in the negative-ionization mode.

Figure S3. Metabolomic composition and structural variation with different treatments (D14). (A) Heatmap of all metabolites detected from different treatments in the positive-ionization mode. (B) Heatmap of all metabolites detected from different treatments in the negative-ionization mode. (C) Relative abundance of top 20 metabolites from all treatments in the positive-ionization mode. (D) Relative abundance of top 20 metabolites from all treatments in the negative-ionization mode. (E) Venn diagram showing the differential metabolites among group comparisons in the positive-ionization mode. (F) Venn diagram showing the differential metabolites among group comparisons in the negative-ionization mode. CK: control; M16: M16 treatment; M44: M44 treatment; Com: combination of M16 and M44; D14: day 14.

Figure S4. Correlations among the top 20 significantly differential metabolites in different comparative groups (M16 vs CK, M44 vs CK, Com vs CK) in two ionization modes. (A) M16 vs. CK in positive mode. (B) M16 vs. CK in negative mode. (C) M44 vs. CK in positive mode. (D) M44 vs. CK in negative mode. (E) Com vs. CK in positive mode. (F) Com vs. CK in negative mode. Orange and blue correspond to positive and negative correlations, respectively. The numbers inside the circles are correlation coefficients ranging from −1 to 1. Circles with numbers were produced only when the correlations between metabolites reached a significant ($p < 0.05$) degree; otherwise, it would be a vacant space at the crossing position of every two metabolites. CK: control; M16: M16 treatment; M44: M44 treatment; Com: combination of M16 and M44; D7: day 7.
