# Supplementary material for: Profiling of Plant Growth-Promoting Metabolites by Phosphate-Solubilizing Bacteria in Maize Rhizosphere
Source: Plants (Basel). 2021 May 27;10(6):1071. doi: 10.3390/plants10061071 (PMC8229199; doi:10.3390/plants10061071)
Supplement: Supplementary file 1 [file plants-10-01071-s001.zip › plants-1169264-supplementary/Supplementary Materials/Table S2. Results of enzyme activities of M16.pdf]

Table S2. Results of enzyme activities of M16.

| Enzymes/Substrates | Results |
|---|---|
| β-Galactosidase | + |
| Arginine dihydrolase | + |
| Lysine decarboxylase | - |
| Ornithine decarboxylase | + |
| Citrate utilization | - |
| H2S production | - |
| Urease/Urea hydrolysis | - |
| Tryptophan deaminase | - |
| Indole production | + |
| Acetoin production | + |
| Gelatinase/Gelatin hydrolysis | - |
| Fermentation/Oxidation of D-glucose | + |
| Fermentation/Oxidation of D-mannitol | + |
| Fermentation/Oxidation of inositol | - |
| Fermentation/Oxidation of sorbitol | + |
| Fermentation/Oxidation of L-rhamnose | + |
| Fermentation/Oxidation of sucrose | - |
| Fermentation/Oxidation of melibiose | - |
| Fermentation/Oxidation of amygdalin | + |
| Fermentation/Oxidation of L-arabinose | + |

"+" means positive reaction, "-" means negative reaction.
