# Supplementary material for: Profiling of Plant Growth-Promoting Metabolites by Phosphate-Solubilizing Bacteria in Maize Rhizosphere
Source: Plants (Basel). 2021 May 27;10(6):1071. doi: 10.3390/plants10061071 (PMC8229199; doi:10.3390/plants10061071)
Supplement: Supplementary file 1 [file plants-10-01071-s001.zip › plants-1169264-supplementary/Supplementary Materials/Table S3. Results of enzyme activities of M44.pdf]

Table S3. Results of enzyme activities of M44.

| Enzymes/Substrates | Results |
|---|---|
| Water (Control) | - |
| Alkaline phosphatase | + |
| Esterase (C4) | + |
| Esterase Lipase (C8) | + |
| Lipase (C14) | - |
| Leucine arylamidase | - |
| Valine arylamidase | - |
| Cystine arylamidase | - |
| Trypsin | W |
| $\alpha$-Chymotrypsin | - |
| Acid phosphatase | + |
| Naphthol-AS-BI-phosphohydrolase | + |
| $\alpha$-Galactosidase | - |
| β-Galactosidase | - |
| β-Glucuronidase | - |
| $\alpha$-Glucosidase | - |
| β-Glucosidase | + |
| N-acetyl-β-glucosaminidase | - |
| $\alpha$-Mannosidase | - |
| $\alpha$-Fucosidase | - |

"+" means positive reaction, "-" means negative reaction, "W" means weakly positive reaction.
