# Supplementary material for: Profiling of Plant Growth-Promoting Metabolites by Phosphate-Solubilizing Bacteria in Maize Rhizosphere
Source: Plants (Basel). 2021 May 27;10(6):1071. doi: 10.3390/plants10061071 (PMC8229199; doi:10.3390/plants10061071)
Supplement: Supplementary file 1 [file plants-10-01071-s001.zip › plants-1169264-supplementary/Supplementary Materials/Table S5. Utilization of carbon sources by M44 to produce acids.pdf]

Table S5. Utilization of carbon sources to produce acids by M44.

Carbon sources	Results	Carbon sources	Results
Control	-	Esculin	+
Glycerol	+	Salicin	+
Erythritol	-	Cellobiose	+
D-arabinose	-	Maltose	+
L-arabinose	+	Lactose	+
D-ribose	+	Melibiose	+
D-xylose	+	Sucrose	+
L-xylose	-	Trehalose	+
D-adonitol	-	Inulin	-
Methyl-β-D-xylopyranoside	-	Melezitose	-
D-galactose	+	Raffinose	+
D-glucose	+	Starch	-
D-fructose	+	Glycogen	-
D-mannose	+	Xylitol	-
L-sorbose	-	Gentiobiose	+
L-rhamnose	-	Turanose	+
Dulcitol	-	D-lyxose	-
Inositol	+	D-tagatose	-
D-mannitol	+	D-fucose	-
D-sorbitol	-	L-fucose	-
Methyl α-D-mannopyranoside	-	D-arabitol	-
Methyl α-D-glucopyranoside	+	L-arabitol	-
N-acetyl-glucosamine	W	Gluconate	-
Amygdalin	+	2-Ketogluconate	-
Arbutin	+	5-Ketogluconate	-

"+" means positive reaction, "-" means negative reaction, "W" means weakly positive reaction.
