# Supplementary material for: Profiling of Plant Growth-Promoting Metabolites by Phosphate-Solubilizing Bacteria in Maize Rhizosphere
Source: Plants (Basel). 2021 May 27;10(6):1071. doi: 10.3390/plants10061071 (PMC8229199; doi:10.3390/plants10061071)
Supplement: Supplementary file 1 [file plants-10-01071-s001.zip › plants-1169264-supplementary/Supplementary Materials/Table S7. The correlation between differential metabolites and plant growth using the Mantel Test.pdf]

Table S7. The correlation between differential metabolites and plant growth using Mantel Test.

Metabolites	Statistic R	*P*-value
N-Acetyl-L-Methionine	0.7352	0.001
Phenylacetaldehyde	0.7284	0.002
Proline-hydroxyproline	0.6684	0.001
N-Acetyl-D-Alloisoleucine	0.6571	0.001
γ-Glutamyl cysteine	0.6371	0.002
DL-α-Tyrosine	0.6317	0.001
Threonine-Leucine	0.6209	0.001
Vanillic acid	0.6181	0.005
γ-Glutamic acid-Leucine	0.6114	0.001
L-Kynurenine	0.5975	0.004
D-Phenylalanine	0.5961	0.006
(±)9-HpODE	0.5951	0.001
Spermine	0.5917	0.002
L-Tyrosine	0.572	0.003
DL-β-Leucine	0.5384	0.002
Boc-β-cyano-L-Alanine	0.5268	0.003
L-Phenylalanine	0.5049	0.004
N-acetyl-L-Ornithine	0.4962	0.003
DL-Norvaline	0.4952	0.002
L-(-)-Methionine	0.494	0.002
5-Hydroxytryptophan	0.4916	0.009
Valine-Serine	0.4806	0.006
Indole	0.4737	0.004
Pyridoxine	0.4735	0.001
Phenylacetyl glycine	0.451	0.007
L-Lysine	0.4494	0.005
Prolyl leucine	0.4375	0.001
L-Histidinol	0.4278	0.002
DL-Lysine	0.4205	0.002
5-Hydroxylysine	0.42	0.022
2-(2-chlorophenyl)-1H-Indole	0.4155	0.003
Cadaverine	0.4082	0.002
α-Linolenic acid	0.353	0.008
Pyridoxamine	0.3444	0.015
Glycerol 3-phosphate	0.333	0.004
N-Acetyl-DL-Tryptophan	0.3215	0.034
L-Methionine sulfone	0.3172	0.03
Proline	0.3099	0.029
L-Alanyl-L-Proline	0.304	0.045
DL-Serine	0.2964	0.026
L-5-Hydroxytryptophan	0.2963	0.04
Phenylalanine-Phenylalanine	0.2874	0.011

Coumarin	0.2819	0.019
L-Homocitrulline	0.2641	0.021
Choline Glycerophosphate	0.2596	0.041
5-oxoproline	0.2505	0.013
N-Oleoyl Dopamine	0.2415	0.013
D-(+)-Proline	0.2169	0.036

Only the metabolites with *P*-value < 0.05 were exhibited in this table.
