# Supplementary figures and images for: Profiling of Plant Growth-Promoting Metabolites by Phosphate-Solubilizing Bacteria in Maize Rhizosphere
Source: Plants (Basel). 2021 May 27;10(6):1071. doi: 10.3390/plants10061071 (PMC8229199; doi:10.3390/plants10061071)

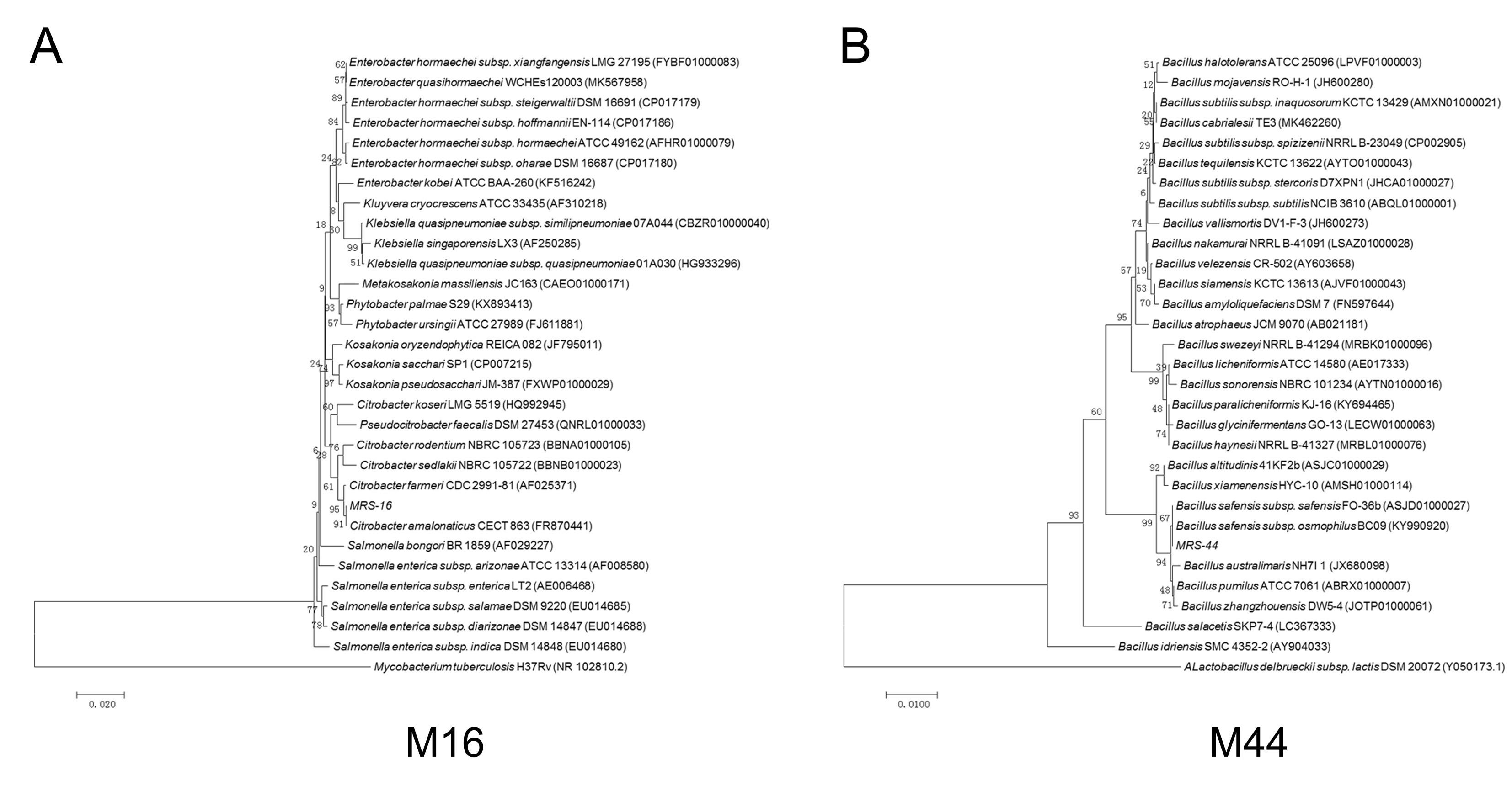

Supplement: Supplementary file 1 [file plants-10-01071-s001.zip › plants-1169264-supplementary/Supplementary Materials/Figure S1. Phylogenetic trees based on 16S rRNA sequencing alignments using the neighbor-joining method.jpg]

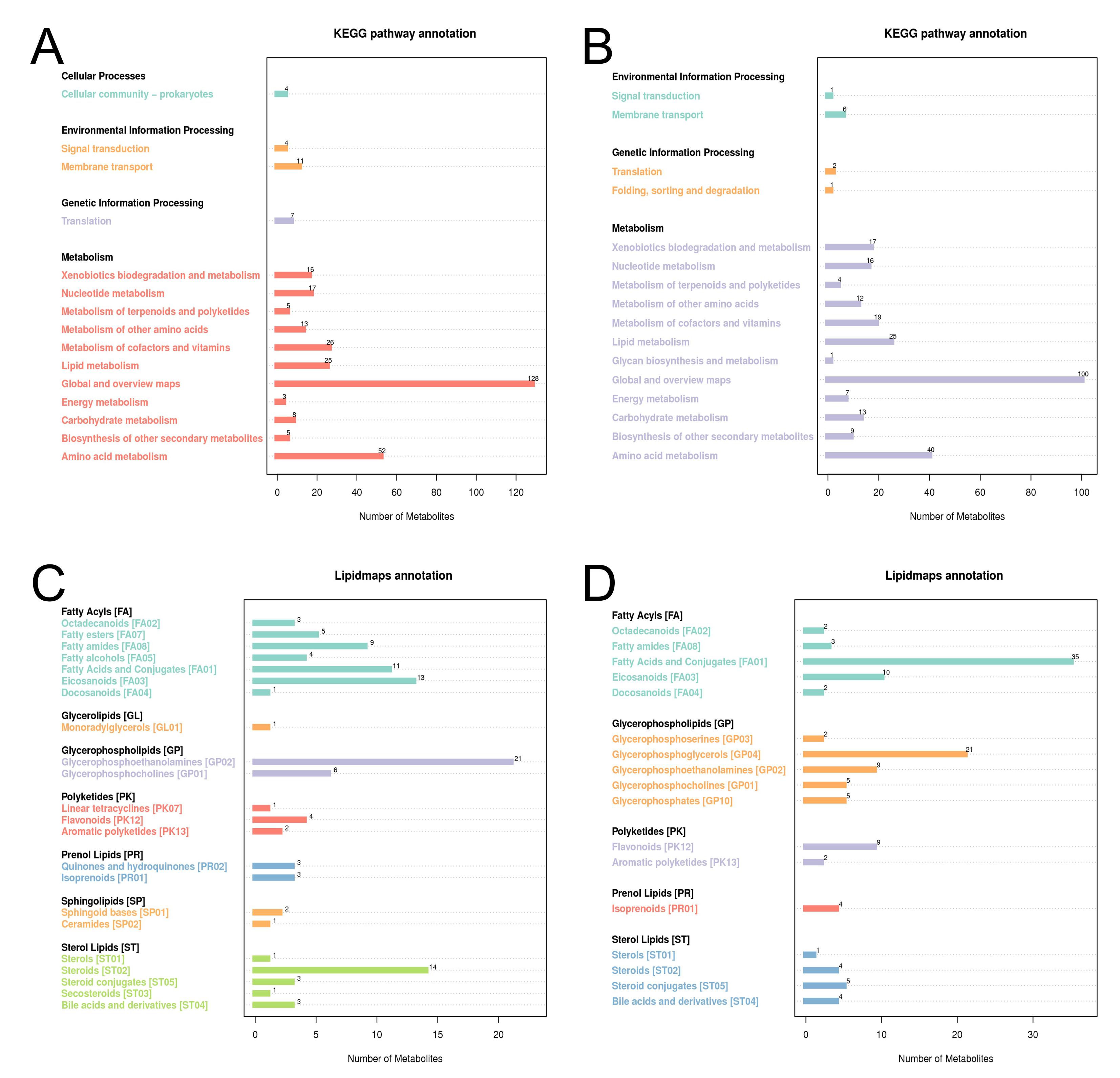

Supplement: Supplementary file 1 [file plants-10-01071-s001.zip › plants-1169264-supplementary/Supplementary Materials/Figure S2. Annotation of metabolites following microbial treatment.jpg]

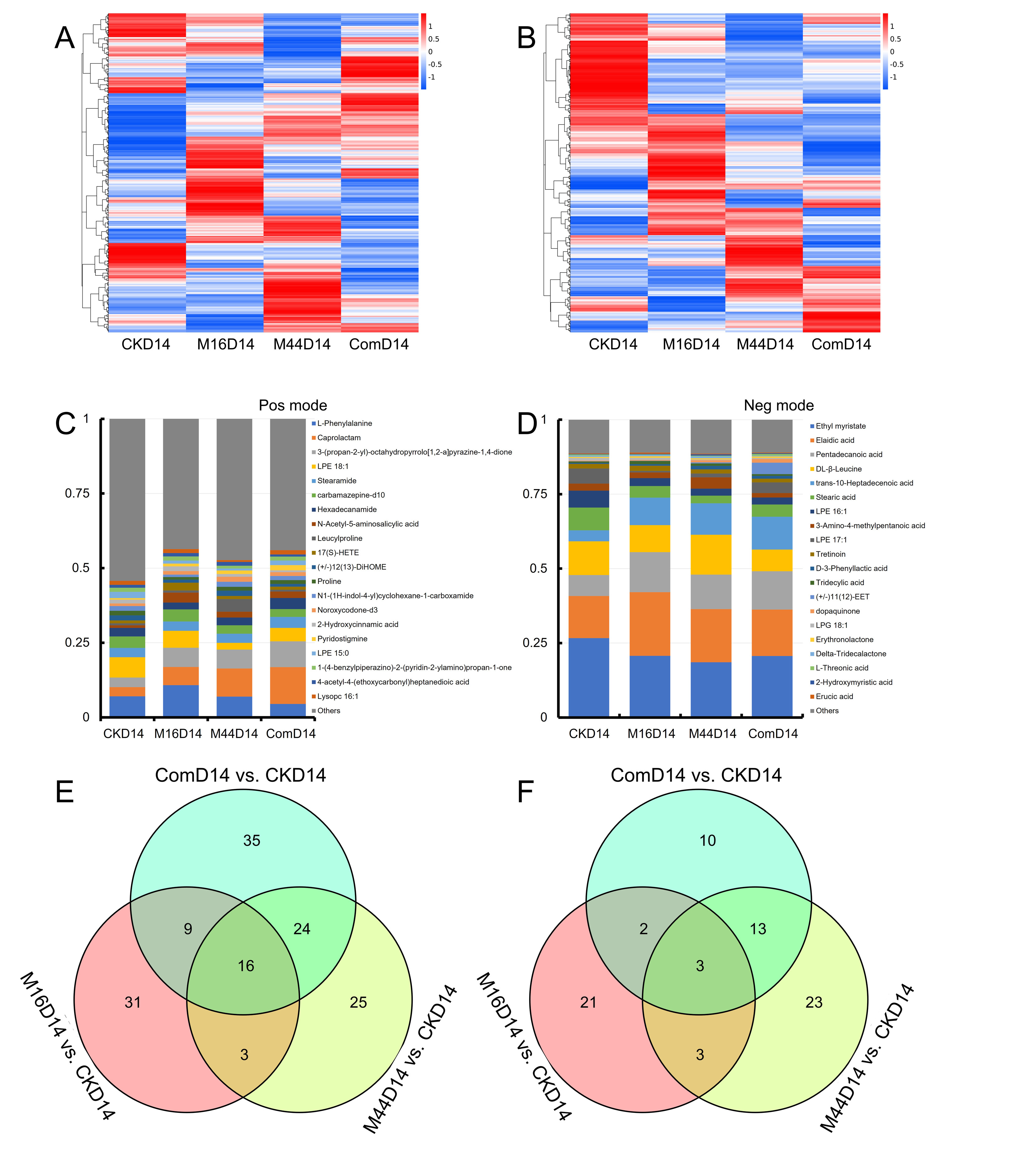

Supplement: Supplementary file 1 [file plants-10-01071-s001.zip › plants-1169264-supplementary/Supplementary Materials/Figure S3. Metabolomic composition and structural variation with different treatments (D14).jpg]

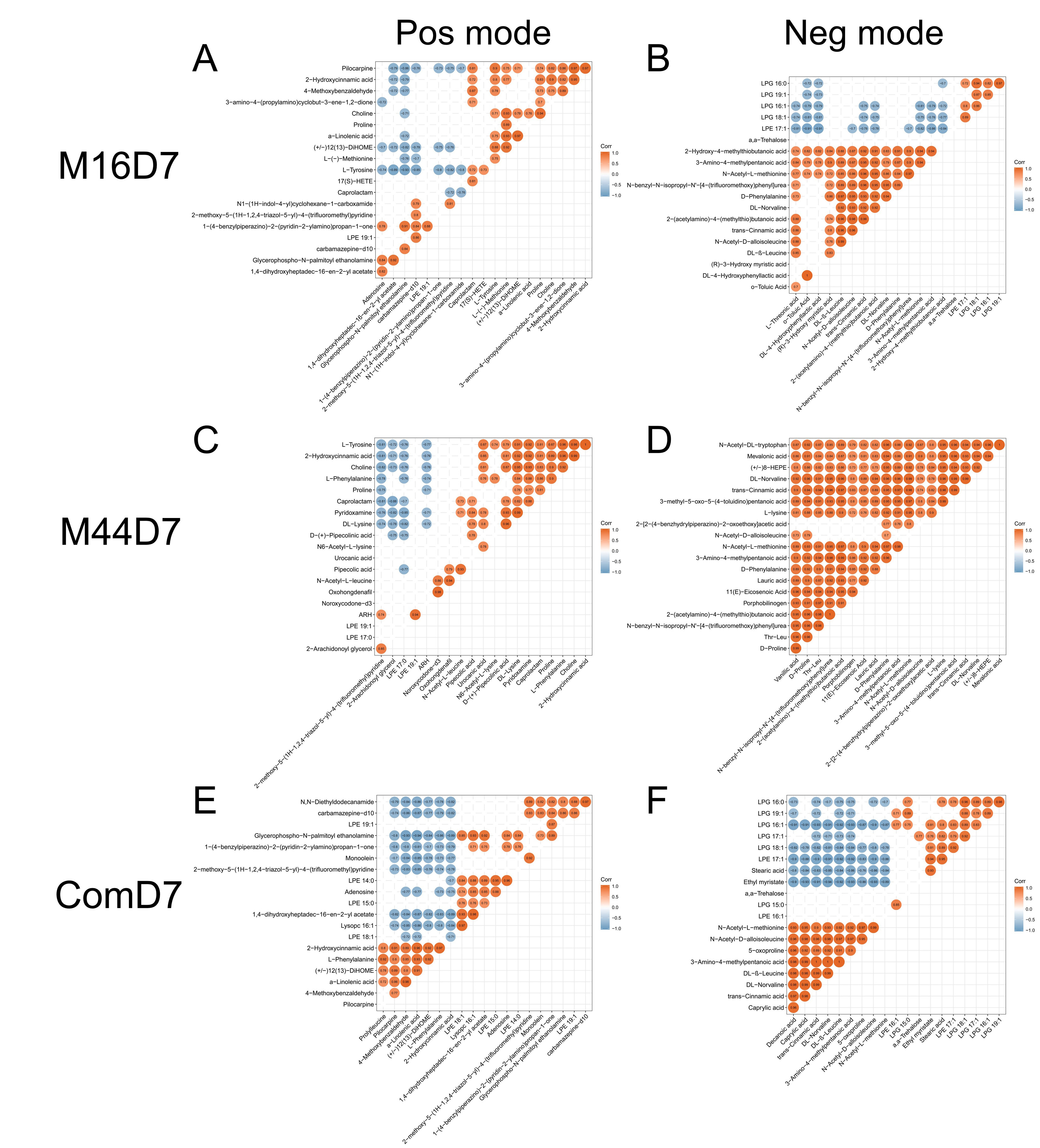

Supplement: Supplementary file 1 [file plants-10-01071-s001.zip › plants-1169264-supplementary/Supplementary Materials/Figure S4. Correlations among the top 20 significantly differential metabolites in different comparative groups (M16 vs CK, M44 vs CK, Com vs CK) in two ionization modes.jpg]
